# A Journey with Elie Metchnikoff: From Innate Cell Mechanisms in Infectious Diseases to Quantum Biology

**DOI:** 10.3389/fpubh.2016.00125

**Published:** 2016-06-16

**Authors:** Fabrice Merien

**Affiliations:** ^1^AUT-Roche Diagnostics Laboratory, Auckland University of Technology, Auckland, New Zealand

**Keywords:** Metchnikoff, innate immunity, infection, inflammation, macrophage, polarization, quantum biology

## Abstract

Many reviews of Elie Metchnikoff’s work have been published, all unanimously acknowledging the significant contributions of his cellular theory to the fields of immunology and infectious diseases. In 1883, he published a key paper describing phagocytic cells in frogs. His descriptions were not just about phagocytes involved in host defense, he also described how these specialized cells eliminated degenerating or dying cells of the host. This perspective focuses on key concepts developed by Metchnikoff by presenting relevant excerpts of his 1883 paper and matching these concepts with challenges of modern immunology. A new approach to macrophage polarization is included to introduce some creative thinking about the exciting emerging area of quantum biology.

## The Journey

Father of natural immunity (1), cellular (2), and modern immunology (3): these are the attributions used by many to recognize and disseminate the genius of Elie Metchnikoff (1845–1916). Numerous scientists in his wake made significant contributions to the development of immunology as a new discipline (4). Originally a zoologist, Metchnikoff started his impressive scientific work as a developmental embryologist under the strong influence of Darwin’s “On the Origin of Species” published in the year 1859. By describing phagocytes and phagocytosis, he discovered one of the most intriguing mechanisms of innate immunity. The centenary of the Nobel award to Elie Metchnikoff and Paul Ehrlich was celebrated in the year 2008. Many reviews of Metchnikoff’s work have been published, all unanimously acknowledging the significant contributions of his comparative approach, embracing “innate” curiosity, careful experimentation, and observation (5–10). Important reviews and books were written and published in English (11, 12) and also in French by his second wife Olga (13). To recognize the importance of Elie’s human and scientific achievements, *The New York Times* presented a full-page analysis of Olga Metchnikoff’s biography of her husband in April 1922 (9). However, Metchnikoff’s cellular theory tends to be overlooked or simply ignored in today’s immunology teaching, perhaps because it was not originally published in English and good quality translations of all his publications are not available. Of Russian origin, Metchnikoff published his work mostly in German until 1888. He then published in French after moving to the *Institut Pasteur* in Paris in France. The use of language translation tools was suggested to me sometime. However, even if they are quite good at getting the meaning of words, accuracy is not offered on a consistent basis when the context is unusual. Most importantly, computer-aided translation cannot solve ambiguities nor render the emotional dimension like a human translator can. In 1883, Metchnikoff published a key paper describing phagocytic cells in frogs (14). His descriptions were not just about phagocytes involved in host defense, he also described how these specialized cells eliminated degenerating or dying cells of the very same host during metamorphosis (from tadpoles to adult frogs): “*The traits of the phagocyte have been retained most completely in the mesoderm where a large number of amoeboid cells occur to ingest the body’s own dead or weak as well as foreign particles such as senescent red blood cells*.” Nowadays, we talk about macrophages scavenging apoptotic cells. It was also the early description of an innate ability of the immune system as being able to recognize self from non-self structures. A full translation (by Fabrice Merien and Kay Vopel) in English (*Investigations of the mesodermal phagocytes of some vertebrates*) and French (*Etude des phagocytes mésodermiques de certains vertébrés*) is given in the Supplementary Material. This perspective focuses on key concepts developed by Metchnikoff, by presenting significant excerpts of his 1883 paper and matching these concepts with challenges in modern immunology. Tracing the history and development of biological sciences, I invite you to journey back in time on a quest packed with the so-called rudimentary methods and tools in the hands of geniuses. We will also travel to a not so distant future by including some thinking about the exciting emerging area of quantum biology.

## Going Beyond the Dichotomic View

**Figure 1:**
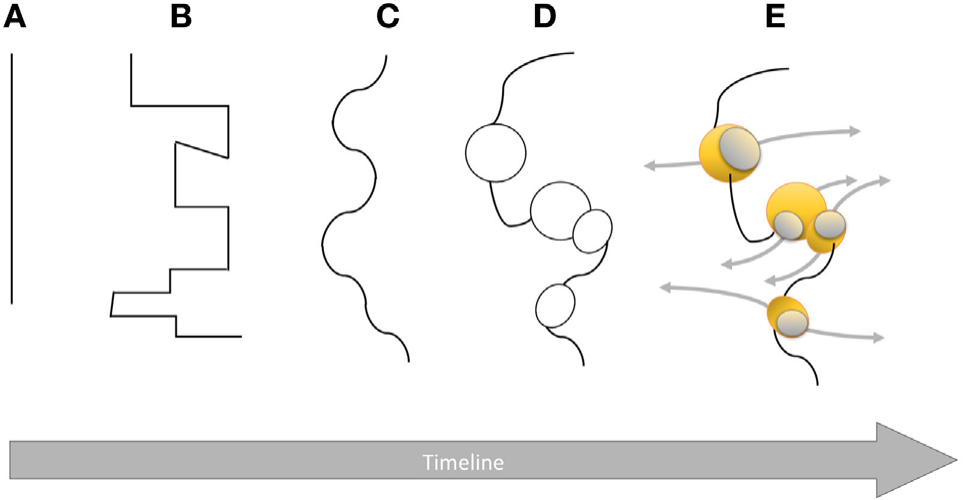
**Graphical representation of the boundary between innate and adaptive immune systems over time from Metchnikoff’s era to modern immunology**. The plain line in **(A)** highlights a clear dichotomy with no connections between the two systems. Then, the line becomes broken **(B)** and sinuous **(C)** evidencing opposite forces from both sides based on scientific breakthroughs. Once researchers have come to a mutually agreed standby point, equilibrium is reached. As more scientific data are available, connections between innate and adaptive systems **(D)** become obvious with areas of similarities or convergence represented by black circles (e.g., complement). This convoluted boundary is not definitive yet as it becomes more permeable **(E)** allowing, in some specific places (yellow spheres), communication (gray arrows) between innate and adaptive immune systems (e.g., memory NK cells, cytokines) introducing more flexibility and complexity.

“*I rather believe that the essence of an inflammation lies in the phagocyte attack of solid pathogenic substances, be it a weakened or dead cell, a bacterium or any other foreign body*.” These words written by Metchnikoff in his 1883 publication clearly put phagocytes in the first line of defense against pathogens. In the same publication, he also wrote: “… *I concluded that the so-called serous inflammation represents an acquired trait, whereas the accumulation of phagocytes constitutes something more primal in the inflammatory response*.” When Metchnikoff studied phagocytes in frogs in 1883, he described which materials were engulfed (cells and dyes) and the morphological changes that accompanied this primordial biological process. In his own words: “*These cells accumulate at the point of inflammation and devour the particles available to them. I have observed, for example, that star-shaped stromal cells feed on red blood cells, carmine and pigment particles. In cases where such cells ingest small numbers of foreign particles, they maintain their star-like shape with only some minor changes in the finest pseudopodia*.” All living organisms from simple prokaryotes to unicellular eukaryotes, invertebrates, and vertebrates protect themselves from pathogens. Defense mechanisms are basic physiological functions that have evolved for survival of the host (15). Phagocytes described by Metchnikoff in frogs can also be found in invertebrates like mollusks. As an example, macrophage-like cells have been found in *Haliotis iris* (black-footed abalone) with other lymphocyte-like cells (16). Understanding the complex interplay between host and pathogen needs a more intricate system than the dichotomic view of the immune response with the innate response, on one side, and the adaptive response, on the other side. Most pathogens have to deal with cellular and humoral effectors (mainly antibodies and complement) of the host immune system. This constant interaction with both innate and adaptive immunities has been triggering ongoing changes in the immune system with more sophisticated and diversified effectors. As with most complex concepts, the human brain tends to use a modular approach that divides the problem into elementary blocks and process them individually. Albeit a useful approach, there is definitely a danger of oversimplification by missing the links connecting those simple blocks. When it comes to the human immune system, we are dealing with a blurring of the classic sharp distinction between innate and adaptive immunities. This paradigm is an oversimplification of a bigger picture that is becoming more and more intricate as more evidence is accumulated (Figure 1). Since Metchnikoff’s era, significant contributions from the scientific community have brought considerable knowledge to the immunology arena, shedding a new light on innate and adaptive immunities that have become more complementary and integrated in the host resistance to infectious diseases.

## Signaling: Catch Me if You Can

Clearance of apoptotic cells is a well-regulated process based on complex signaling mechanisms. Senescent or damaged cells undergoing apoptosis are cleared by macrophages and dendritic cells. Different sensing signals (“find me” and “eat me”) and their cognate receptors are involved in the recognition and processing of apoptotic cells during the phagocytic response (17). Only cells exposing a “eat me” signal are engulfed. Phosphatidylserine (PtdSer) is the most likely “eat me” signal candidate. Proteins called “scramblases” are irreversibly activated to expose PtdSer to the outer leaflet of the plasma membrane (18). Interestingly, apoptosis is not always immunologically silent. Apoptotic cells that are not fully engulfed promote inflammation and activate the immune system by releasing uncleared corpses from the ruptured plasma membrane (17). “Eat me” signals are the key to silent phagocytosis to maintain tissue homeostasis, whereas “find me” signals provide a chemotactic guidance to find apoptotic cells (19). Understanding of these signaling pathways is in its infancy. Deciphering them will tackle some challenging questions for immunologists. All these “find me” and “eat me” signals are promising therapeutic targets for the treatment of multiple immunological disorders and cancer diseases. As an example, defect in exposing important “eat me” signals, such as the surface calreticulin, may dramatically impair phagocytic clearance of cancer cells (20). Also, dying cancer cells going through immunogenic cell death (ICD) produce damage-associated molecular patterns (DAMPs) that interact with dendritic cells and other cells of the immune system (21). These discontinuous molecular patterns displayed on dying or dead cells are in line with the contemporary criterion of continuity (22), where an effector immune response is caused by a strong antigenic discontinuity instead of a clear cut difference between endogenous and exogenous antigens as accepted in the conventional self/non-self consensus.

## The Many Facets of Macrophages

Interestingly, Metchnikoff already foresaw some sort of communication happening between remote events during the inflammation process: “… *we must assume the existence of a living chain that links the particulate stimulus with the blood vessel. This chain will enable a reaction of blood phagocytes even if these are far away from the inflammatory stimulus*….” In 1883, signaling molecules (and their receptors) enabling cells like macrophages to respond to essential signals in a multicellular organism had still to be discovered. To perform complex functions from immune defense to repair and development, macrophages undergo different states of polarization (23). M1 and M2 pathways in macrophages are relevant in infectious pathogenesis, resistance to infection, and chronic evolution of infectious diseases (24, 25). Canonical biomarkers have been described for both M1 and M2 macrophages in clinical investigations. However, as most infectious diseases do not come with these polarized macrophages, this concept has been revisited based on the comparison of transcriptomes from activated monocytes and macrophages as reported by Ka et al. (26). As observed by these authors, the concept of macrophage polarization has already been shifting to a more complex and dynamic vision of macrophage activation that goes beyond the dichotomic M1/M2 classification. It seems that we have a whole spectrum of polarized macrophages or signatures, hence the need for a different approach to describe this process in human infectious diseases. Depending on gene expression, it is easy to visualize a modulation (26) of the information carried by macrophages. The classification has become increasingly confused and complex (27) with new classifications replacing older ones without addressing the uniqueness of each and every macrophage (28). Distinct macrophage populations exist that are all subjected to many different stimuli that modify their gene expression. Macrophages submitted to classical (Th1) pathway are activated by IFN-γ or LPS, whereas macrophages going through the alternative (Th2) pathway are activated by cytokines, such as IL-4, IL-10, or IL-13 (29). However, it is rather difficult to find specific sets of genes that could be used as a signature of activated macrophages. Membrane-expressed receptors or reactivity to cytokines (e.g., IL-4 and alternatively activated macrophages) do not match well with coexpressed sets of genes (28).

In physics, spectral modulation can be applied to the wavelengths of light. If we think of each and every macrophage as a unique wavelength, then all these macrophages become part of a spectrum (27), as we would get white light by combining all the wavelengths of the visible spectrum. Now the question is: how do we connect physics to cellular immunology? Interestingly, quantum biology may be helpful here. Let us start by following a non-representationalist concept defined by Bitbol and Luisi (30) in their study about autopoiesis. Applied to immunology, the relevant concept is the information provided by local environmental conditions for keeping a functional macrophage (cell unit). This unit is made of a vast number of variants (polarized macrophages) in which the changes (e.g., surface receptors, ligands, produced cytokines) are determined in relation to environmental disturbances (e.g., inflammation, infection). The activity of these variants is governed by the local (micro)environment itself and also bacteria as a result of host–pathogen interactions. We know that there is more to this: stimulated by specific signals, polarized macrophages interact with their close environment, and in turn modify it. Recently, it has been hypothesized that epigenetics could be the link between environment and macrophage phenotype by enhancing its diversity and plasticity (23). As a dualist representation, the M1/M2 dichotomy, easy as it is to visualize, may be a bit too simple (31). M1 and M2 come with subcategories, which in turn can be subdivided into distinct cell subsets providing more variants. However, the M1/M2 concept, originally developed in C57Bl/6 and BALB/c mice, does not work well with other animal species (28). Instead of having a finite number of variants that can be easily counted, we have an infinite number of polarized macrophages. All possible states exist simultaneously. As an example, using a microarray approach, Ka et al. (26) showed that M1/M2 polarization is transient, suggesting that macrophage programing is a very dynamic mechanism over time (e.g., acute vs. late infection). Is it the observation or measurement itself that cause the polarized macrophages to be limited to a single or a few possibilities? By asking this question, we have just entered the emerging field of quantum biology. Superposition is a principle of quantum theory that describes a challenging concept about the nature and behavior of matter and forces at the subatomic level. The principle of superposition claims that while we do not know what the state of any object is, it is actually in all possible states simultaneously, as long as we do not observe or measure. Does quantum superposition play a role in macrophage polarization? Is quantum superposition involved in the continuum of functional states (24) of macrophages? The answer might be weirder than we think. Some hypotheses are still speculative, and the question of whether or not quantum physics plays a decisive role in macrophage biology remains unanswered. However, we should not forget that quantum biology underpins life at some molecular level: quantum mechanics (or quantum tunneling to be exact) beautifully explains spontaneous DNA mutations and how enzymes work (32). There is actually no link between the many faces of macrophage activation and quantum biology, hence the need for abstraction to start building a research framework. Theories are important in immunology as demonstrated by Pradeu et al. with the antigenic discontinuity theory (33). When it comes to the formulation of new theories, all fields of science (not only immunology) are required to provide predictive statements. The link between macrophage activation and quantum biology may be bold, but it has the merit to open an exciting field that could contribute to the advancement of biological sciences. This is exactly what Metchnikoff did when he proposed his theory on phagocytosis. At the end of the nineteenth century, Metchnikoff’s theory on phagocytosis was just coming of age and already had to face numerous detractors. It also encouraged open-minded scientists to investigate and come with new interpretations. Quantum biology is still taking its first steps, and we should not underestimate its importance. Let us see what the future will bring. As a pioneer and visionary, Metchnikoff would have enjoyed this new challenge.

## The Challenge of Teaching Immunology

Before starting a lecture about Metchnikoff’s great achievements, I like starting my immunology classes by asking a simple question to my students: “Close your eyes, empty your mind, forget your immediate surroundings, and describe how a hospital laboratory was at the end of the nineteenth century in Europe?” Interestingly, it is a challenging exercise for young people who are so used to automate multiplexing high-throughput medical analyzers. At that time, commands like “press the start button” or “perform a calibration test” were out of this world when quality control, accuracy, precision, and traceability still had to be introduced. From the observation of biological specimens with eyes to molecular methods, the sophistication of scientific techniques, from empirical to experimental, has come a long way. Starting with a simple microscope with a light collector and other basic methods, Elie Metchnikoff successfully performed a scientific study of infection and immunity.

Recent advances and technologies in immunology have stimulated new approaches to understanding the complex nature of the immune response. As an example, the antigenic discontinuity theory proposed by Pradeu et al. (22) introduces changes that are affecting the theoretical framework of immunity, bringing our understanding of innate immunity under new light. The “speed of change” (22) in immunology is unprecedented, boosted by advances in genomics, proteomics, and other -omics technologies. Because of its association with many other subjects (e.g., microbiology, hematology, histology, cytology, transfusion medicine, and molecular diagnostics) taught at universities, immunology is becoming a popular topic. Describing a new mechanism or identifying a new component in the already well-described B, T, or NK cell pathways goes beyond the fame of theory when translation to control and cure of diseases (e.g., cancer and autoimmune diseases) is possible.

Defining “immunology” can be a challenge by itself, if one wishes to understand the complexity of immune systems in the context of the evolutionary history of invertebrates and vertebrates. It is widely accepted that the “innate” immune system predates the “adaptive” immune system. Both systems encompass specific effector cells and molecules that are largely integrated as complementary partners in vertebrates such as humans.

Defining an “immunologist” can also be a difficult task. What immunologists do on a daily basis depends on whether they work for a clinical laboratory, a pharmaceutical company, or a university. Teaching immunology at universities is becoming more challenging, given the pace of scientific breakthroughs in biology and medicine. In a world where time constraint is a reality faced by all lecturers, teaching programs and training curricula still need to be constantly updated to include the latest insights. We must be careful, however, not to forget the basics taught by the forefathers of immunology. Elie Metchnikoff, the “father of natural immunity” was one of our forefathers along with Louis Pasteur and Robert Koch with their breakthroughs in bacteriology. Remembering Metchnikoff’s contributions and modern advances (34) is the best way to understand the important concepts of today’s immunology and trigger curiosity and engagement of students. The reading of his study published in 1883 (see translations in the Supplementary Material) immediately makes us ask more questions: what, where, when, and how? Most relevant is the word “how” as illustrated by Brian Cox in his *Wonders of Life* (35): “*The word ‘how’ transforms it, and provides a significant and important insight into the mind of a scientist. Let us find out by studying nature, developing theories and testing those theories against our observations of the living world, how life can be fully explained by the laws of physics and chemistry, as it surely must be*.” Elie Metchnikoff would have fully acknowledged this statement.

## Author Contributions

FM was involved in the conception of this work. The author critically drafted it and submitted the final version for publication.

## Conflict of Interest Statement

The author declares that the research was conducted in the absence of any commercial or financial relationships that could be construed as a potential conflict of interest.
