# Supplementary material for: A Journey with Elie Metchnikoff: From Innate Cell Mechanisms in Infectious Diseases to Quantum Biology
Source: Front Public Health. 2016 Jun 16;4:125. doi: 10.3389/fpubh.2016.00125 (PMC4909730; doi:10.3389/fpubh.2016.00125)
Supplement: Supplementary file 1 [file presentation_1.pdf]

Biodiversity Heritage Library

http://www.biodiversitylibrary.org

Biologisches Zentralblatt

Leipzig [etc.] VEB Georg Thieme [etc.]

http://www.biodiversitylibrary.org/bibliography/6184

Bd. 3 (1883-1884): http://biodiversitylibrary.org/item/27783

Pages 560-565

Contributed by: MBL WHOI Library

Sponsored by: MBL WHOI Library

http://www.biodiversitylibrary.org/pdf3/005050700027783

Biologisches Centralblatt.

Unter Mitwirkung von

Dr. M. Reess

Professor der Botanik

und

Dr. E. Selenka

Professor der Zoology

herausgegeben von

Dr. J. Rosenthal

Professor der Physiology in Erlangen

Dritter Band.

1883—1884.

Mit 23 Holzschnitten.

Erlangen, 1884.

Verlag von Eduard Besold.

Biologisches Centralblatt

unter Mitwirkung von

Dr. M. Reess

Professor der Botanik

und

Dr. E. Selenka

Professor der Zoology

herausgegeben von

Dr. J. Rosenthal

Professor der Physiology in Erlangen

---

24 Nummern von je 2 Bogen bilden einen Band. Preis des Bandes 16 Mark. Zu beziehen durch alle Buchhandlungen und Postanstalten.

III. Band. 15. November 1883. Nr. 18.

Inhalt: **Neelsen**, Neue Ansichten ueber die Systematik der Spaltpilze.—**Dewitz**, Ueber das verschiedene Aussehen der gereizten und ruhenden Druesen im Zehenballen des Laubfrosches.—**Metschnikoff**, Untersuchungen ueber die mesodermalen Phagocyten einiger Wirbeltiere.—**Gottschau**, Ueber die Nebennieren der Saeugetiere.

Investigations of the mesodermal phagocytes of some vertebrates

By Dr Elie Metchnikoff (Elias Metschnikoff)

Pages 560—565

Translated by Fabrice Mérien and Kay C. Vopel, AUT Auckland University of Technology.

I have recently[1] used the term phagocytes to describe all cells capable of ingesting and, if possible, digesting solid food. The traits of the phagocyte have been retained most completely in the mesoderm where a large number of amoeboid cells occur to ingest the body's own dead or weak as well as foreign particles such as senescent red blood cells. In pathology, comprehensive material has already been accumulated that describes such ability in white blood cells; however, it has not yet been accepted that the uptake of foreign particles and the decomposition of these particles (e.g., red blood cells) represent the processes of ingestion and digestion, respectively. Studies of invertebrates and, in particular, evidence for the digestive role of amoeboid mesoderm cells in sponges together with the finding that in *Bipinnara*, *Phyllirhoë* and others, such cells perhaps function as digestive organs, led to the conclusion that intracellular digestion must also take place in the mesoderm of vertebrates.

The tail of batrachians must be considered as a particularly suitable object to verify this conclusion. It holds a large number of amoeboid cells right from the first stages of its atrophy. These cells contain fragments of nerve and muscle fibers. It only takes a fragment of an atrophic tail pulled to pieces in blood serum or eye fluid to free numbers of these cells. Left undisturbed for a while, these cells will spread a quantity of fine pseudopodia, so that they resemble *Actinophrys* and other Heliozoa. Only in the cases of a few larvae of *Bombinator* was I able to observe this behavior on intact tails. These observations revealed that, as metamorphosis starts, the amoeboid cells that accumulate next to tail muscles first gradually engulf and then digest fragments of muscle fibers. The ingested muscle fragments maintain their normal shape for some time before their transverse striation disappears and they finally disintegrate into roundish refracting particles.

I usually found a considerable number of very similar amoeboid cells in the visceral cavity of metamorphosing batrachians. These cells, however, only contained roundish

[1] Untersuchungen über die intrazelluläre Verdauung bei wirbellosen Tieren in Claus, Arb. aus dem Zool. Inst. zu

particles but no muscle fragments. I believe that I can reasonably conclude that the phagocytes responsible for the atrophy of the tail first reach the visceral cavity and then are transferred into the lymph or blood system.

The atrophy of the gills is not so easy to observe. However, during metamorphosis these organs, too, contain a sufficient number of well-fed phagocytes.

Hence we can conclude that phagocytes play an equally important and active role in the radical metamorphosis of batrachians than that I have demonstrated for *Bipinnaria* and *Auricularia*. Additional support for my observations comes from pathologists: they found that the so-called active atrophy of muscles and nerves involves cells with similar functions, the migratory cells.

To confirm that the phagocytes of vertebrates, as those of invertebrates, are capable of feeding on parasitic bacteria, I have induced an artificial septicemia in frogs by injecting foul blood under the skin. The white blood cells of the diseased animals included variable numbers of mobile and immobile bacilli, which were contained inside vacuoles. I found a particularly large number of these bacilli in the phagocytes of the spleen, which confirms the commonly held view of pathologists, that the white blood cells usually transport hardly soluble or insoluble particles to the spleen. Such findings seem to support the assumption that the spleen functions as a prophylactic organ, removing the source of inflammation, similar to my assumptions for nematocalyxes of Plumularids. This assumption is supported by the findings of Bacelli[2]; that fresh spleen juice dissolves coagulated proteins. The common observation that many animals from which the spleen is removed live on untroubled is fully in line with the view that the spleen has no crucial physiological role but functions as an organ that fights inflammatory particles (in particular, bacteria). Hence**,** it would be of interest to compare the resistance (to infection) of animals with spleen removed to that of normal animals, but it should not be ignored that similar prophylactic roles can be ascribed to other organs, for example the lymphatic glands and the bone marrow.

Since my observations on invertebrates lead me to conclude that the essential and genealogically original mechanism of an inflammation lies in the accumulation of

[2] Studien über die Funktion und die Pathologie der Milz. *Virchow's Archiv* Bd. 51. 1870. S. 141.

phagocytes for the purpose of devouring inflammatory particles, I, first and foremost, had to raise the question of how the findings on vertebrates could be brought into line with this conclusion. I identified the tail fin of *Triton* and other batrachian larvae as the most suitable study object. An inflammation can quite easily be observed after touching a spot at the tail fin of a *Triton cristatus* larva with a small piece of lunar caustic (Höllenstein or infernal stone), immediately followed by rinsing with sodium chloride solution. Here, the usual symptoms with respect to blood vessels are much less pronounced than those found in frog larvae, perhaps because the tail fin of a *Triton* larva possesses thinner vessels that the considerably larger blood cells struggle to penetrate. However, *Triton* larvae are a convenient study object to obtain reliable information about the reactive role of stroma cells in an inflammation. These cells accumulate at the point of inflammation and devour the particles available to them. I have observed, for example, that star-shaped stroma cells feed on red blood cells, carmine and pigment particles. In cases where such cells ingest only small numbers of foreign particles, they maintain their star-like shape with only some minor changes in the finest pseudopodia. In contrast, in cases where these cells feed more foreign particles, the fine pseudopodia retract and the cell more or less loses its star-like shape and shows an increased mobility. My observations, by all means, revealed that a sharp distinction between so-called stationary or star-shaped and wandering stroma cells does not exist. Finally, the well-fed cells retract most or all pseudopodia after they have changed into a roundish or oval clump.

Hence, I arrived at the conclusion that the stroma cells of *Triton* larvae tail fins must definitely be considered as phagocytes and that they react as such during inflammation. In several cases I was able to observe the multiplication by division of such cells in inflamed larvae. However, this process, which can easily be observed in all stages on a living animal by following chromosome alignment patterns during mitosis (« Kernfiguren »), is too rare to deserve a significant role in the inflammation process.

In the case of tadpoles, the migration of white blood cells plays a much more important role as in *Triton* larvae, although stroma cells do contribute to the inflammation process. My observations support the view of those researchers who, as recently as Von Recklinghausen, assume an active migration of white blood cells. I have observed such typical migration, from capillaries containing stagnant blood, in a few instances. This

migration was initiated by the spreading of pseudopodia: something that white blood cells of invertebrates usually do under stagnant conditions. Here, the phagocytes also accumulated at the centre of the inflammatory lesions, that I created in tadpoles with a thin glass capillary containing cobalt green or carmine, and engulfed everything within reach. They usually maintained this condition for several days and weeks. In cases where some well-fed phagocytes died, these were eaten by other phagocytes, so that it was not uncommon to observe a large mononuclear cell that contained one or two dead, enucleated phagocytes. Truly giant cells, that are polynuclear plasmodia, did not usually form. In all of my studies of amphibian larvae, I have observed such cells only in rare cases.

From my experiences, I must add the following: first, in several cases I noticed that the greatest emigration of phagocytes in inflamed tadpoles did not occur in the immediate vicinity of the glass capillary but at some distance from it. Secondly, I never observed a considerable accumulation of transudates but instead always an accumulation of phagocytes. From the latter I concluded that the so-called serous inflammation represents an acquired trait, whereas the accumulation of phagocytes constitutes something more primal in the inflammatory response.

All of my experiences with both invertebrates and amphibians are hardly in line with the current theory of the inflammatory process, which states that the essence of this process lies in a disease of the blood vessel wall. I rather believe that the essence of an inflammation lies in the phagocyte attack of solid pathogenic substances, be it a weakened or dead cell, a bacterium or any other foreign body. In invertebrates, where plenty of phagocytes are available, this response proceeds without contribution from the blood vessel wall. In vertebrates, however, the wall of the blood vessel contributes to the inflammatory process in places that the extra-vascular phagocytes are unable to reach. I believe that the involvement of white blood cells is mediated by stroma and endothelial cells: it is well known that the cells of the endothelium retain, to some degree, the ability to move and contract. The stimulus first triggers a response in the phagocytes of the stroma, which, as described above, are far from being passive during an inflammation but more or less retract their pseudopodia. This response can affect the intact (living) wall of the endothelium in a way that not only supports the active emigration of blood phagocytes but also the passive diapedesis of red blood cells. Following this hypothesis, we must assume the existence of a

living chain that links the particulate stimulus with the blood vessel. This chain will enable a reaction of blood phagocytes even if these are far away from the inflammatory stimulus as in the case, for example, of keratitis. This chain consists of three links: 1) stroma phagocytes, 2) endothelial cells of the blood vessel wall and 3) white blood cells.

If this briefly described viewpoint is false and the theory which states that the essence of an inflammation lies in the lesion of the vessel wall and the subsequent passive extravasation of the blood elements is correct, then one would expect**,** in cases where the stimulus is located within the blood itself, that an emigration would still proceed as the blood elements move away from the stimulus. This would be the case in bacterial diseases in which the parasites live in the blood. However, we did not observe such extravasation when, as in the above discussed frog septicemia, for example, the blood vessels were stimulated by a large number of mobile bacilli. The white blood cells, without crossing the wall of the blood vessel, attack and if possible eliminate the pathogenic bacteria in the blood. Furthermore, in the case of a recurrent attack, enormous numbers of spirilla circulate in the blood for several days, "without, apparently, affecting at all the blood flow, or heart and blood vessels"[3], which anyway aligns better with the above developed hypothesis than with the current inflammation theory. Also, it is known that a blood clot, occurring outside the blood vessel, causes an inflammatory reaction, that is, attracts phagocytes. A simple thrombus, however, which directly affects the wall of the blood vessel, does not necessarily cause an inflammation, perhaps because a sufficient number of phagocytes circulates in the blood. Put in this context, one can view the battle between phagocytes and particulate stimuli inside the blood as a kind of "haemitis".

[3] Cohnheim, Vorles. üb. allgemeine Pathologie. 2. Auflage. Bd. I. 1882. S. 475.

Etude des phagocytes mésodermiques de certains vertébrés

Par le Dr Elie Metchnikoff (Elias Metchnikoff)

Pages 560-565

Traduit par Fabrice Mérien et Kay Vopel, AUT Auckland University of Technology.

J'ai récemment[1] utilisé le terme 'phagocyte' pour décrire les cellules capables d'ingérer et, si possible, digérer des aliments solides. La plupart des traits caractéristiques des phagocytes sont analysables dans le mésoderme où un grand nombre de cellules amiboïdes sont observées ingérant des « substances » mortes ou anormales du corps (endogènes) et des éléments étrangers (exogènes). En pathologie, des données complètes décrivant une telle fonction chez les cellules blanches du sang sont déjà abondamment disponibles. Toutefois, il n'est pas encore accepté que l'absorption de particules étrangères et la dégradation de ces particules (par exemple les cellules rouges du sang) représentent respectivement les processus d'ingestion et de digestion. Les résultats d'une étude comparative sur des éponges comme *Bipinnara*, *Phyllirhoë* et d'autres encore, m'ont permis d'observer, au niveau du mésoderme, que des cellules amiboïdes exercent une fonction de digestion. Ce faisant, j'ai estimé essentielle de valider ou d'invalider l'hypothèse selon laquelle la digestion intracellulaire pouvait être une fonction exercée par des cellules stromales présentes dans le mésoderme des vertébrés.

La queue des larves de batraciens peut être considérée comme un organe particulièrement utile à la vérification de cette hypothèse. Elle contient un grand nombre de cellules amiboïdes dès les tout premiers stades de son atrophie. Ces cellules contiennent des fragments de nerfs et de fibres musculaires. Il suffit d'un fragment de queue atrophiée, réduit en petits morceaux dans du sérum ou de l'humeur aqueuse, pour récupérer, en grande quantité, ces cellules libérées de leur matrice. Laissées au repos pendant un certain temps, ces cellules produisent un certain nombre de fins pseudopodes qui les font ressembler à *Actinophyris* et autres *Heliozoa*. Ce n'est que dans le cas de quelques larves de *Bombinator* que j'ai pu observer ce comportement avec des queues intactes. Ces

observations ont révélé que, à partir du moment où commence la métamorphose, les cellules amiboïdes qui s'accumulent près des muscles de la queue absorbent graduellement puis digèrent les fragments de fibres musculaires. Les fragments de muscle ingérés conservent leur forme normale pendant un certain temps avant que leur aspect strié ne s’estompe, puis finalement se désintègrent en particules arrondies réfractant la lumière.

J’ai généralement trouvé un nombre considérable de cellules amiboïdes très similaires dans la cavité viscérale de batraciens en cours de métamorphose. Ces cellules, cependant, ne contenaient que des particules arrondies et pas de fragments de muscles. Je crois que je peux raisonnablement conclure que les phagocytes responsables de l'atrophie de la queue atteignent d'abord la cavité viscérale à partir de laquelle ils vont ensuite rejoindre la lymphe ou le système sanguin.

L'atrophie des branchies n'est pas aussi facile à observer. Cependant, lors de la métamorphose, ces organes aussi contiennent des phagocytes « bien nourris » en grand nombre.

Par conséquent, nous pouvons conclure que les phagocytes jouent un rôle tout aussi important et actif dans la métamorphose fondamentale des batraciens, comme j'ai pu le démontrer pour *Bipinnaria* et *Auricularia*. Une source d’arguments supplémentaires en appui de mes observations a été générée par les pathologistes: ils ont constaté que l'atrophie dite active des muscles et des nerfs implique des cellules aux fonctions similaires, les cellules migratrices.

Pour confirmer que les phagocytes de vertébrés, comme ceux des invertébrés, sont capables d’ingérer des bactéries parasites, j'ai provoqué une septicémie artificielle chez des grenouilles en leur injectant du sang putréfié sous la peau. Les cellules blanches du sang des animaux malades contenaient des quantités variables de bacilles mobiles et immobiles, qui étaient enfermés à l'intérieur de vacuoles. J'ai trouvé un nombre particulièrement élevé de ces bacilles dans les phagocytes de la rate, ce qui confirme l’idée acceptée par les pathologistes que les cellules blanches du sang transportent habituellement des particules à peine solubles ou insolubles dans la rate. Ces résultats semblent confirmer l'hypothèse que la rate fonctionne comme un organe prophylactique supprimant les sources d'inflammation, tout comme j'ai pu le constater avec les nématothèques de Plumularides. Cette hypothèse

est étayée par les conclusions de Bacelli[2] qui montrent que le suc de rate fraiche dissout les protéines coagulées. En ayant recours à des métazoaires sains maintenus dans des conditions expérimentales contrôlées, il avait été noté que la splénectomie ne se traduisait par aucune anomalie cliniquement détectable: cet organe ne serait-il donc fonctionnel que chez des métazoaires auxquels seraient inoculés des particules sources d'inflammation (en particulier, les bactéries). Il serait donc intéressant de comparer la résistance de tels animaux avec celle d'animaux normaux, sans oublier qu'on doit attribuer des rôles prophylactiques semblables à d'autres organes tels que, par exemple, les ganglions lymphatiques et la moelle osseuse.

Depuis que mes observations sur les invertébrés m'ont amené à la conclusion que le mécanisme essentiel et originel de l'inflammation réside dans l'accumulation des phagocytes chargés de dévorer des particules inflammatoires, j'ai d'abord et avant tout dû m'interroger sur la façon de mettre ce mécanisme en relation avec mes résultats obtenus chez les vertébrés. J'ai choisi la nageoire caudale du Triton et autres larves de batraciens comme objet d'étude le plus approprié. Une inflammation peut être assez facilement observée après avoir frotté une zone de la nageoire caudale d'une larve de *Triton cristatus* avec un peu de « caustique lunaire » (*note du traducteur : encore dénommé pierre infernale par les auteurs du 17e au 19e siècle, c'était un caustique des plus violents composé de nitrate d'argent fondu et fabriqué avec des « cristaux de lune » et de « l'esprit de nitre »*), immédiatement suivi d'un rinçage avec une solution de chlorure de sodium. Dans ces conditions, les symptômes habituels notés au niveau des vaisseaux sanguins sont beaucoup moins prononcés que ceux trouvés dans les larves de grenouille, probablement du fait que la nageoire caudale d'une larve de Triton possède des vaisseaux sanguins plus fins, où les cellules géantes du sang peinent à pénétrer. Cependant, les larves de Triton restent un objet d'étude idéal pour obtenir des informations fiables sur la réactivité des cellules du stroma lors de l'inflammation. Ces cellules s'accumulent sur le lieu de l'inflammation et dévorent les particules à leur portée. J'ai observé, par exemple, que les cellules étoilées du stroma se nourrissent de cellules rouges du sang, de carmin et de particules de pigment. Dans les cas où de telles cellules n'ingèrent que de petites quantités de particules étrangères, elles conservent leur forme étoilée avec uniquement quelques changements mineurs au niveau des pseudopodes les plus fins. En revanche, dans les cas où elles se nourrissent en

abondance de particules étrangères, les pseudopodes se rétractent et la cellule perd plus ou moins sa forme étoilée et montre une mobilité accrue. Mon observation a révélé, sans doute possible, qu'il n'y a pas de différences nettes entre les cellules dites ancrées au stroma en forme d'étoile, et les cellules mobiles du stroma. Enfin, les cellules bien nourries rétractent la plupart ou la totalité de leurs pseudopodes après avoir pris une forme arrondie ou ovale.

Par conséquent, j'arrive à la conclusion que les cellules du stroma de la nageoire caudale de la larve de Triton doivent définitivement être considérées comme des phagocytes et en tant que tels qu'elles « réagissent » pendant l'inflammation. Dans plusieurs cas, j'ai pu observer la multiplication par division de ces cellules dans des larves sujettes à une inflammation. Toutefois, ces divisions cellulaires facilement observables à toutes les étapes sur un animal vivant en suivant les profils d'alignement des chromosomes lors de la mitose (« Kernfiguren »), sont trop rares pour mériter de se voir attribuer un rôle important au cours d'un processus inflammatoire de courte durée.

Dans le cas des têtards, la migration des cellules blanches du sang joue un rôle beaucoup plus important que dans les larves de Triton, bien que les cellules du stroma contribuent au processus inflammatoire. Mon observation soutient le point de vue de chercheurs qui, comme récemment Friedrich Daniel von Recklinghausen, ont mis en évidence la migration active des cellules blanches du sang. J'ai observé dans quelques cas cette migration typique dans des capillaires en verre contenant du sang stagnant. Cette migration était initiée par le développement de pseudopodes ; quelque chose que les cellules blanches du sang d'invertébrés ont l'habitude de faire dans ces conditions. Chez les têtards, quand je crée une inflammation avec un capillaire de verre mince contenant du vert de cobalt ou du carmin, les phagocytes s'accumulent au niveau de cette blessure et ils absorbent tout ce qu'ils peuvent atteindre. Ils demeurent généralement dans cet état durant plusieurs jours et semaines. Dans les cas où certains phagocytes bien nourris meurent, ils sont à leur tour engloutis par d'autres phagocytes, de telle manière qu'il n'est pas rare d'observer une grande cellule mononuclée contenant un ou deux phagocytes énucléés morts. D'authentiques cellules géantes, comme des plasmodies polynucléaires, ne sont généralement pas observées. Dans toutes mes études sur les larves d'amphibiens, je n'ai observé de telles cellules que dans de rares cas.

A propos de mes expériences, je dois ajouter ce qui suit. Tout d'abord, dans plusieurs cas, j'ai remarqué que la plus grande migration des phagocytes chez les têtards soumis à une inflammation n'a pas eu lieu dans le voisinage immédiat du capillaire de verre, mais à une certaine distance. Deuxièmement, je n'ai jamais observé d'accumulation importante de transsudats, mais toujours une accumulation de phagocytes. De cette dernière observation je conclus que l'inflammation dite séreuse est une caractéristique tardive de la réponse inflammatoire, alors que l'accumulation de phagocytes constitue quelque chose de plus précoce dans la réponse.

L'ensemble de mes expériences avec les invertébrés et les amphibiens n'est guère en accord avec la théorie actuelle de l'inflammation, qui stipule que la cause du processus se situe dans une atteinte de la paroi des vaisseaux sanguins. Je pense plutôt que la cause de l'inflammation réside dans l'attaque par les phagocytes de substances pathogènes solides, qu'il s'agisse de cellules « affaiblies » ou mortes, d'une bactérie ou de toute autre substance étrangère. Chez les invertébrés, où beaucoup de phagocytes sont disponibles, cette réponse inflammatoire se produit sans aucune contribution de la paroi des vaisseaux sanguins. Chez les vertébrés cependant, la paroi du vaisseau sanguin contribue au processus inflammatoire dans les endroits qui ne peuvent pas être atteints par les phagocytes extravasculaires constitutivement présents dans le stroma. Je crois que cette participation des cellules blanches du sang est médiée par les cellules du stroma et de l'endothélium ; il est bien connu que les cellules de l'endothélium conservent, dans une certaine mesure, leur capacité à se déformer et se contracter. Le premier stimulus déclenche une réponse des phagocytes du stroma, qui comme décrit plus haut ne restent pas passifs pendant l'inflammation, et rétractent plus ou moins leurs pseudopodes. Cette réponse peut affecter la paroi intacte (vivante) de l'endothélium d'une façon qui non seulement facilite la migration active des phagocytes du sang mais aussi la diapédèse passive de cellules rouges du sang. En continuant sur la lancée de cette hypothèse, nous devons supposer l'existence d'une chaîne vitale qui relie le stimulus au vaisseau sanguin. Cette chaîne permettra une réaction en chaîne des phagocytes du sang, même si ceux-ci sont éloignés du stimulus inflammatoire, comme c'est le cas, par exemple, lors d'une kératite. Cette chaîne se compose de trois éléments : 1) les phagocytes du stroma, 2) les cellules endothéliales de la paroi du vaisseau sanguin et 3) les cellules blanches du sang.

Si ce point de vue brièvement décrit est faux, mais si la théorie qui affirme que la cause de l'inflammation est liée à une lésion de la paroi du vaisseau et une extravasation passive des éléments du sang est correcte, on pourrait alors s'attendre à ce que dans les cas où le stimulus est situé dans le sang même, une migration se produise, permettant aux éléments sanguins de s'éloigner du stimulus. Ce serait le cas des maladies bactériennes dans lesquelles les parasites vivent dans le sang. Cependant, nous n'avons pas observé d'extravasation quand par exemple, comme dans la septicémie discutée plus haut, les vaisseaux sanguins étaient stimulés par un grand nombre de bacilles mobiles. Les cellules blanches du sang, sans traverser la paroi du vaisseau sanguin, attaquent et si possible éliminent les bactéries pathogènes dans le sang. De plus, en cas d'attaque récurrente, de très nombreux spirilles circulent dans le sang pendant plusieurs jours "sans apparemment affecter la circulation sanguine, ou le cœur et les vaisseaux sanguins"[3], ce qui est plus en accord avec l'hypothèse développée plus haut, comparée à la théorie actuelle de l'inflammation. Ainsi est-il bien connu qu'un caillot de sang se produisant à l'extérieur d'un vaisseau sanguin, cause une réaction inflammatoire qui attire les phagocytes. Cependant, un simple thrombus qui affecte directement la paroi du vaisseau sanguin ne produit pas nécessairement d'inflammation, probablement parce qu'un nombre suffisant de phagocytes circule dans le sang. Dans ce contexte, on peut envisager la bataille entre les phagocytes et les particules à l'intérieur du sang comme une sorte d'inflammation du sang.

[1] Untersuchungen über die intrazelluläre Verdauung bei wirbellosen Tieren in Claus, Arb. aus dem Zool. Inst. zu Wien Bd. V, Heft II, S. 141 ff. Taf. XIII, XIV

[2] Studien über die Funktion und die Pathologie der Milz. Virchow's Archiv Bd. 51. 1870. S. 141.

[3] Cohnheim, Vorles. üb. allgemeine Pathologie. 2. Auflage. Bd. I. 1882. S. 475.
